# Peak Expiratory Flow Predicts Incident Dementia in a U.S. Sample of Older Adults

**DOI:** 10.1093/geroni/igab046.3508

**Published:** 2021-12-17

**Authors:** Patrick Donahue, Qian-Li Xue, Michelle Carlson

**Affiliations:** 1 Johns Hopkins Bloomberg School of Public Health, Baltimore, Maryland, United States; 2 Johns Hopkins University School of Medicine, Baltimore, Maryland, United States; 3 Johns Hopkins University, Baltimore, Maryland, United States

## Abstract

Dementia is an increasingly important public health problem with known vascular contributors. Respiratory function, measured by peak expiratory flow (PEF), may be a novel modifiable risk factor in reducing the risk of dementia along the vascular pathway. We investigated the association between PEF and incident dementia in older adults from the National Health and Aging Trends Study (NHATS). Using NHATS criteria, participants were categorized as having or not having probable incident dementia during NHATS Rounds 2-4, spanning three years. Of 3,622 participants with available PEF and covariate data, 543 (15.0%) had incident cases of dementia. Quartile of baseline PEF was analyzed as a predictor of incident dementia using logistic regression models, while controlling for several health and sociodemographic covariates. The fourth quartile of PEF had statistically significantly decreased odds of incident dementia when compared to the first PEF quartile (OR = 0.27; 95% CI [0.19, 0.40]). Significantly reduced odds of incident dementia were found when comparing the third and second PEF quartiles to the first PEF quartile, as well (OR = 0.35; 95% CI [0.26, 0.47] and OR = 0.62; 95% CI [0.48, 0.80], respectively). These relationships were dose-dependent so that increasing PEF quartile levels were more protective against incident dementia. PEF may be considered as an easily administered, low-cost measure of respiratory function and a possible screening tool for dementia risk. Improving PEF may reduce dementia risk through vascular mechanisms (e.g., increased blood circulation and brain oxygenation). Future research should explore these potential causal pathways between PEF and dementia.

